# The role of artificial intelligence in burn assessment, complication diagnosis, and outcome prediction: a narrative review

**DOI:** 10.1093/burnst/tkaf071

**Published:** 2025-10-30

**Authors:** Punit Bhattachan, Zachary Ricciuti, Fadi Khalaf, Marc G Jeschke

**Affiliations:** Department of Surgery, McMaster University, 1280 Main St W, Hamilton, ON, L8S 4L8, Canada; Centre for Burn Research, Hamilton Health Sciences, 20 Copeland Ave, Hamilton, ON, L8L 2X2, Canada; David Braley Research Institute, Hamilton Health Sciences, 20 Copeland Ave, Hamilton, ON, L8L 2X2, Canada; Centre for Burn Research, Hamilton Health Sciences, 20 Copeland Ave, Hamilton, ON, L8L 2X2, Canada; David Braley Research Institute, Hamilton Health Sciences, 20 Copeland Ave, Hamilton, ON, L8L 2X2, Canada; Department of Medical Sciences, McMaster University, 1280 Main St W, Hamilton, ON, L8S 4L8, Canada; Centre for Burn Research, Hamilton Health Sciences, 20 Copeland Ave, Hamilton, ON, L8L 2X2, Canada; David Braley Research Institute, Hamilton Health Sciences, 20 Copeland Ave, Hamilton, ON, L8L 2X2, Canada; Department of Biochemistry and Biomedical Sciences, McMaster University, 1280 Main St W, Hamilton, ON, L8S 4L8, Canada; Department of Surgery, McMaster University, 1280 Main St W, Hamilton, ON, L8S 4L8, Canada; Centre for Burn Research, Hamilton Health Sciences, 20 Copeland Ave, Hamilton, ON, L8L 2X2, Canada; David Braley Research Institute, Hamilton Health Sciences, 20 Copeland Ave, Hamilton, ON, L8L 2X2, Canada; Department of Medical Sciences, McMaster University, 1280 Main St W, Hamilton, ON, L8S 4L8, Canada; Department of Biochemistry and Biomedical Sciences, McMaster University, 1280 Main St W, Hamilton, ON, L8S 4L8, Canada

**Keywords:** Artificial intelligence, Machine learning, Deep learning, Burn injury, Complications, Sepsis, Inhalation injury, Acute kidney injury, Predictive models

## Abstract

Burn injury remains a major global health challenge, causing an estimated 180 000 deaths annually. The marked heterogeneity in burn severity, complications, and outcomes highlights the need for more objective and efficient evaluation strategies. Artificial intelligence (AI) has emerged as a promising approach to support clinical decision-making and improve patient care in this field. In this narrative review, we summarize the growing applications of AI in burn care, including the assessment of burn depth and total body surface area, monitoring of wound healing, prediction of postburn complications, and estimation of clinical outcomes. AI-based models have demonstrated strong performance in automating wound assessment, optimizing fluid resuscitation, and predicting complications such as sepsis, inhalation injury, and acute kidney injury. Furthermore, AI-driven prediction of mortality risk and hospital length of stay has shown potential to inform early interventions and improve resource allocation. Despite encouraging progress, most studies to date rely on small, single-center datasets and limited model validation, underscoring the need for larger, multi-institutional efforts, and standardized data sharing. Integrating AI into burn management holds great promise for enhancing diagnostic precision, forecasting outcomes, and personalizing treatment strategies. As these technologies advance, clinician familiarity and collaboration with AI tools will be critical to fully realize their potential in transforming burn care.

Highlights

- Artificial intelligence (AI)-driven models based on machine learning and deep learning have demonstrated strong performance in burn assessment, early detection of complications, and outcome prediction. These tools hold considerable potential for integration into routine burn care and clinical decision-making.
- Extensive clinical and omics datasets provide valuable resources for developing robust AI models that can enhance diagnostic accuracy and deepen understanding of burn pathophysiology. However, large-scale prospective studies and randomized clinical trials are still needed to validate and standardize these applications.
- Integrating AI into burn management can significantly improve the quality and efficiency of care. Nonetheless, ethical considerations surrounding patient privacy, data security, and algorithmic transparency must be carefully addressed to ensure responsible implementation.

## Background

Burns are one of the leading causes of injury in humans, with an age-standardized incidence rate of ~110 per 100 000 people [1]. Burn injuries claim around 180 000 lives per year due to subsequent complications such as hepatic steatosis, systemic inflammation, cardiac dysfunction, sepsis, cachexia, and multi-organ failure [2]. The evidence surrounding the use of artificial intelligence (AI) in burn care is beginning to grow; however, it is still unclear whether recent findings sum together to support the future implementation and integration of AI technologies into burn clinics. Generally, AI algorithms are designed to unravel hidden patterns in large datasets and perform a multitude of repetitive tasks to answer questions incapable of being addressed via conventional statistical means [3]. The process of designing, building, and utilizing an AI tool requires significant research and planning and includes multiple phases, each defined by a series of steps [4]. AI is divided into machine learning (ML) and deep learning (DL) subfields [5, 6] based on their methodological characteristics and the process associated with generating these models (Table 1).

**Table 1:** Differences between machine learning and deep learning

	Machine learning (ML)	Deep learning (DL)
1.	Common in clinical studies.	Less common in clinical studies.
2.	ML uses statistical methods of training.	DL uses artificial neural network for training.
3.	ML uses structured small datasets such as vital measurements, lab data, omics data, etc.	DL uses large unstructured dataset such as clinical notes, films, burn images, etc.
4.	Interpretability of data is easier and transparent in clinical settings.	It is like a black box. The interpretability of data is low.
5.	ML modeling is faster.	DL is slower and needs GPU.
6.	Clinical biases are often recognizable.	Clinical biases are hidden and difficult to detect.

AI has multiple emerging applications in burn care and research, which can be broadly categorized into burn severity and wound management, postburn complications, and postburn outcomes, with various models tested and the most effective models summarized in Table 2. The burn severity and wound management domain includes subspecialties such as burn size calculation, burn depth assessment, and wound healing assessment. These applications primarily utilize clinical image data of burns as input features into DL models, which then perform tasks to provide the desired clinical assessment. AI tools in this area have demonstrated utility in estimating %TBSA, guiding fluid resuscitation, monitoring progress of wound healing, and more. These tools have the potential to become vital pieces of burn care by enabling faster and more accurate clinical decision-making. On the other hand, AI applications may even extend to aiding in the management of postburn complications such as sepsis diagnosis, drug treatment, and the detection of inhalation and kidney injury. Indeed, these applications can also be harnessed to enhance prognostic capacity by improving mortality prediction and length of hospital stay for burn patients. However, it is important to recognize that the use of AI within these contexts has inherent limitations. Burn severity and wound management assessment mainly digest clinical images as input features within DL models. While comprehensive model training may mitigate inaccuracies, the major limitation of DL models is interpretability. DL models act like a black box in that we understand the input layer and output layer, but the processes of multiple hidden layers in between them always remain obscure, posing challenges for clinical validation and trust. Similar drawbacks are found in ML models, as they commonly consume numerical datasets as input features which can then be vulnerable to biases, thus limiting performance and interpretability.

**Table 2:** Summary of burn topics, AI model tested, and the best AI model for each application in burn care

	Burn topics	Subsections	AI models tested	Best AI model
1.	Burn severity and wound healing assessments	AI models for burn size calculation	U-Net, Mask R-CNN, PSPNet, DeepLabV3+, RestNet101	DeepLabV3+
		AI models for burn depth assessment	CNN, joint-task DL models, CNN-BAM	CNN-BAM
		AI models for wound healing assessment	ML-SATA, CNN-BAM	CNN-BAM
2.	Postburn complications	Sepsis diagnostics and drug treatment	ML-MILO (DNN, LR, Naïve Bayes, KNN, SVM, RF, and GBM), ANN	KNN, ANN
		Inhalation and kidney injury	ML-GBM, KNN, LR, SVM, RF, DNN	GBM, DNN
3.	Postburn outcomes	Mortality predictions	Naïve Bayes, DT, SVM, BP, extreme boosting model, RF, GLRM, KNN, MLP, AdaBoost	ANN
		Length of hospital stay prediction	LR, RF, LightGBM, XBGBoost	XBGBoost

Importantly, AI tasks are not completely independent of human input. While these tools may be designed to perform many clinical tasks attached to the job of a burn care physician, they are not designed to replace the physician. Moreover, these should be seen as strong supplements rather than artificial replication of human responsibilities, requiring that the burn care providers develop a foundational understanding of AI tools to effectively integrate them into clinical tasks and ultimately improve patient care.

**Figure 1:**
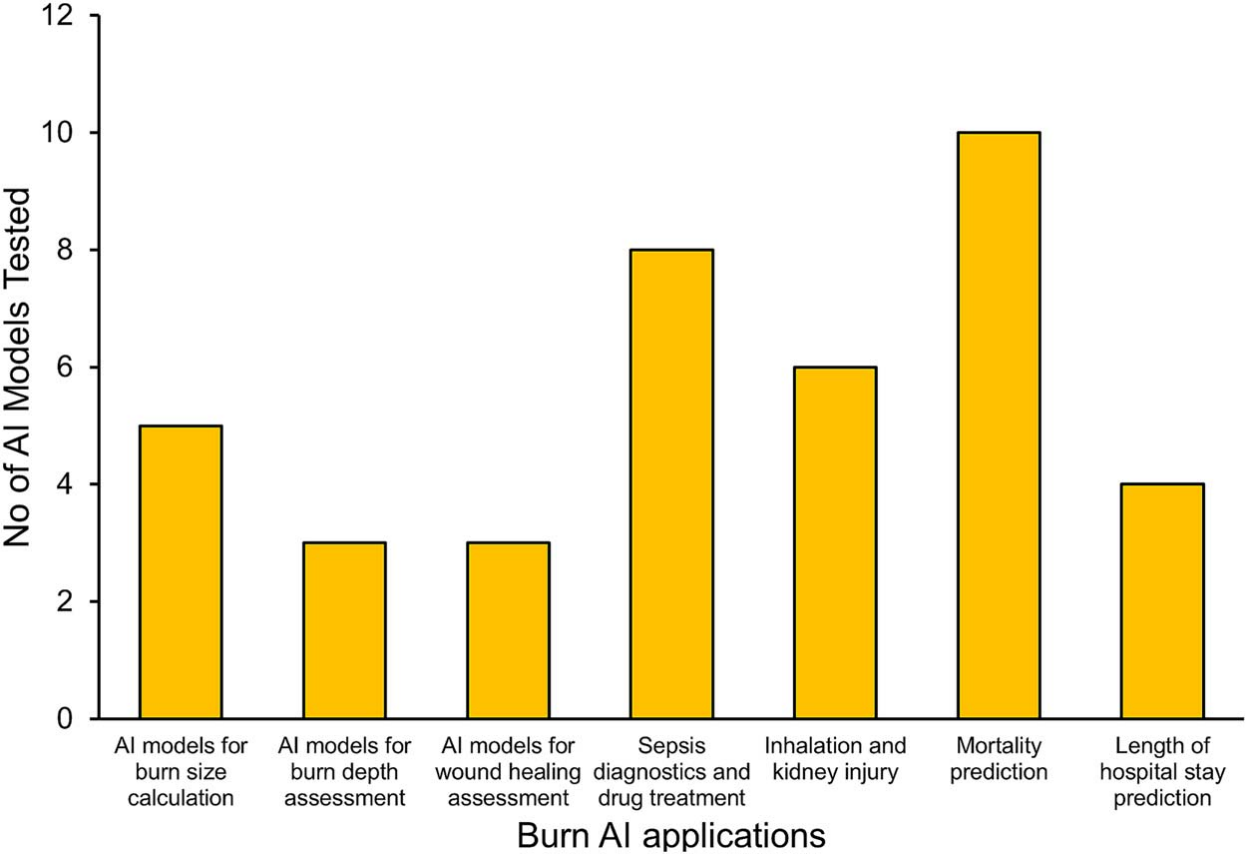
Number of AI models tested in burn care and research; the bar plot displayed the number of AI models tested on the y-axis and burn AI applications on the x-axis

In this review, we aim to explore the application of AI and its subfields, namely ML and DL, and discuss not only the various AI models currently applied within burn care research but also the efficacy of these models and their potential in transforming burn care and patient outcomes. We also discuss important ethical considerations when collecting and utilizing data from burn patients for AI models, as well as highlight future perspectives in combining AI models with high-throughput statistical approaches, such as omics technologies. A comprehensive literature search was conducted using the queries “Burn AND Artificial Intelligence,” “machine learning in burns,” “deep learning in burns,” and “AI in burns” on both PubMed and Google Scholar databases. Only articles published in English language were considered. Figure 1 summarizes the results of our search and the number of AI models tested in burn care and research.

## Review

### Burn severity and wound healing assessment

**Figure 2:**
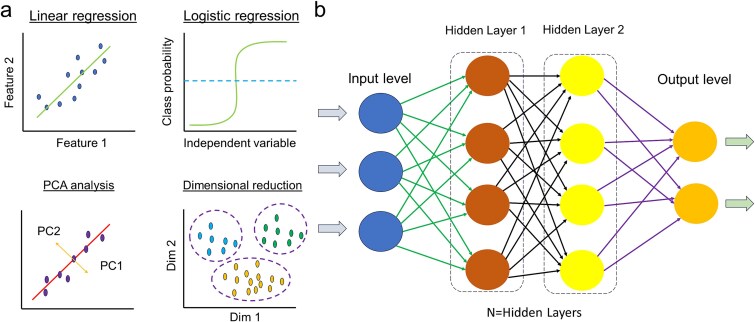
Machine learning (ML) and deep learning (DL) tools; (**a**) ML methods; ML uses different statistical methods such as linear regression, logistic regression, PCA, dimensional reduction, etc., for the analysis; (**b**) neural architecture; DL uses artificial neural networks for the analysis

Traditional methods for evaluating burn severity and progression often rely on subjective clinical judgment, which can lead to variability in diagnosis and treatment decisions. AI-driven models (Figure 2) have the potential to improve accuracy, consistency, and efficiency in burn assessment by analyzing complex patterns in medical data. The following sections explore the role of AI in three key areas: burn size estimation, burn depth classification, and wound healing assessment.

### Artificial intelligence models for burn size calculation

Burn injuries present with considerable variability in wound size, depending on the type and severity of the injury. For instance, scald burns lead to rapid spread of energy, increasing the total area of the burn, whereas flame-induced burns cause an immediate deep injury, limiting its total area but enhancing the degree of the burn [2]. Consequently, an accurate calculation of the burn size is a critical aspect of burn care. In fact, the total body surface area (TBSA) covered by a burn injury serves as one of the main prognostic factors for mortality and length of stay (LOS) following a burn injury [7]. Multiple studies have identified cutoff values for burn sizes that are associated with the greatest risk of morbidity and mortality, allowing practitioners to identify their most vulnerable patients and adding further importance to accurate calculation of burn wound size [8, 9]. Indeed, severe burns are classified as >10% TBSA in older adults (≥60 years), >20% TBSA in adults (≥18 and <60 years), and >30% TBSA in pediatrics (<18 years) [2]. Moreover, these severe burn injuries trigger a profound and prolonged hypermetabolic response, which places the patient at greater risk of negative outcomes such as sepsis, cachexia, and multi-organ failure [2]. The Wallace Rule of Nines is considered the gold standard for estimating burn size, as it assigns a standardized percentage to each region of the body regardless of an individual’s weight or body shape [10]. However, calculating burn size by this method can be challenging and prone to error due to the irregular and variable shapes of burn wounds as well as size and weight differences among patients [11].

Recent advancements in AI have demonstrated significant potential in improving the accuracy of burn size estimation, addressing the limitations of traditional methods like the Wallace Rule of Nines. In 2021, Chang *et al*. conducted a study utilizing two different types of DL models (U-Net and Mask region-based convolutional neural network [R-CNN]) with the aim of improving the accurate calculation of burn wound size [12]. The Mask R-CNN model of %TBSA calculation had a smaller average deviation (0.115% TBSA) from the ground truth—a pixel-based calculation of affected %TBSA—than five burn surgeons who provided their respective estimations based on images of the burn wounds. The improved accuracy of the Mask R-CNN model has important clinical relevance, given that the Parkland formula for calculating resuscitation fluid volume depends on precise estimation of the burned %TBSA. Over and underestimation of volume needed for fluid resuscitation can lead to negative consequences such as pulmonary complications and renal failure [13]. Therefore, improved accuracy in burn size calculation can optimize this aspect of care for burn patients and reduce the risk of complications. Following these findings, the same research group reported the use of multiple DL architectures to estimate %TBSA. In their investigation, U-Net, pyramid scene parsing network (PSPNet), DeepLabV3+, and Mask R-CNN DL models with encoders ResNet101 were used for training and testing the data. Among these, the DeepLabV3+ model demonstrated slightly better performance in determining %TBSA burned; however, all models performed favorably and provided evidence for the introduction of AI models in supporting consistent and accurate assessments of burn wounds [14]. The key difference in both studies was the use of the hand and palm area, respectively, to estimate the %TBSA of burn area by the DL method, and it was revealed that estimation of %TBSA based on the palm area was relatively better. In a related advancement, Xu *et al.* developed a joint-task DL model with an accompanying mobile application to assess %TBSA burned using color images captured by a smartphone [15]. This framework was tested on 1340 burn wound images and achieved an R^2^ value (quantifies the disparity between estimated and actual TBSA) of 0.9136, suggesting the DL model had a high degree of accuracy. Given the wide variety of smartphones available, each with different camera resolutions and image qualities, it is unlikely that all clinicians will use the same device to capture burn images. Therefore, establishing general guidelines for smartphone camera specifications and standardized image acquisition methods is essential to ensure consistent implementation of the current AI tool.

### Artificial intelligence models for burn depth assessment

While accurate burn size estimation is essential at admission to ensure proper resuscitation and initial management, burn wounds typically take several weeks to heal, progressing through distinct phases: hemostasis, inflammation, proliferation, and remodeling. The healing process is heavily influenced by burn depth, which is categorized into four levels: first-degree (superficial thickness), second-degree (partial thickness), third-degree (full thickness), and fourth-degree burns [16]. Beyond its role in burn size estimation, AI has also demonstrated significant potential in evaluating burn depth, a key determinant of wound healing and a crucial factor in guiding treatment decisions.

Despite technologies such as laser Doppler imaging (LDI), infrared thermography, and photoacoustic imaging [17–20], burn depth assessment remains prone to misdiagnosis, even among experienced burn surgeons, where the reported accuracy is only between 64% and 76% [21]. Burn wounds undergo significant changes within the first week following injury, meaning the initial assessment of burn depth may not stand true several days after the injury. Furthermore, similar to burn size calculation, burn depth assessment is an area which could potentially benefit from the incorporation of AI models. To provide rapid and accurate determination of burn depth, Thatcher *et al*. used multispectral images of burns as input data and compared the ability of three CNN architectures and an ensemble to automatically highlight areas of nonhealing burn regions [22]. The results showed that the ensemble model was the most effective predictor with 81% sensitivity, 100% specificity, and 97% positive predictive value (PPV). Additionally, the ensemble’s sensitivity and PPV increased in a sigmoid shape throughout the first week postburn, demonstrating its accuracy in spite of dynamic wound changes. Similarly, Xu *et al*. tested a joint-task DL model, which was highlighted in the latter section, on its accuracy of burn depth segmentation—i.e. the segmentation of burn regions into three burn degrees: superficial partial thickness, deep partial thickness, and full thickness [15]. The average dice coefficient (considers true positive, false positive, and false negative values) for burn depth segmentation was 85.12%, highlighting the ability of this model to accurately assess both burn depth and %TBSA burned. In a separate study, a new boundary attention mapping (BAM) algorithm was combined with CNN to predict burn depth via images of the burn wounds and compared against the commonly used LDI [23]. The CNN-BAM model burn segmentation achieved a 91.6% accuracy, 78.2% sensitivity, and 93.4% specificity, supporting their hypothesis that the model would have comparable efficacy to LDI. Importantly, the CNN-BAM model is designed to be applied in a mobile application format, therefore creating an accessible and inexpensive burn depth assessment tool with equivalent accuracy to LDI. Interestingly, older adult populations were included in this study, while the majority of current AI burn studies did not include them. This is particularly important given that older adult burn patients experience unique physiological challenges and significantly poorer outcomes compared to younger individuals, likely due in part to metabolaging—a progressive decline in metabolic flexibility associated with aging [24].

The growing body of evidence supporting AI-driven burn size estimation highlights its potential to enhance accuracy and consistency in clinical assessments. By overcoming the limitations of traditional methods, AI models offer a more precise and efficient approach to determining the extent of a burn, ultimately improving patient care and treatment planning. However, beyond burn size and depth estimation, AI is also proving to be a valuable tool in assessing wound healing. Given that the progression of burn wounds depends on factors such as depth, tissue damage, and individual healing responses, AI-driven models are now being explored to evaluate and predict wound healing outcomes more effectively.

### Artificial intelligence models for wound healing assessment

Early wound closure following a burn injury is a critical factor in patient outcomes, contributing to lower mortality rates, reduced hypertrophic scarring, and shorter hospital stays [25–29]. Despite it being a fundamental aspect of wound care, there is a lack of validated, objective methods to assess wound closure, making it challenging to compare the effectiveness of new treatment approaches. Fortunately, with advancements in AI, recent studies have explored innovative approaches to enhance wound healing assessment, offering potential insights to guide future treatment strategies.

A study by Ethier *et al.* utilized computer vision and AI, specifically an algorithm termed Skin Abnormality Tracking Algorithm (SATA), which consisted of a CNN-BAM model. The model was employed to assess and quantify the burn wound healing within one patient across a total of 8 weeks, six in clinic and two once discharged via remote monitoring [30]. Following the study period, the authors concluded that the SATA assessment correlated with those assessments conducted by a clinician, with the added benefit of producing more quantitative data than typically seen in clinical wound healing measures. SATA must be tested on larger sets of burn images from cohorts with greater heterogeneity; however, the preliminary results suggest this is a promising technology with potential to improve burn wound assessments.

### Postburn complications

Beyond skin damage, severe burn patients often face additional life-threatening complications, including inhalation and kidney injury and a heightened risk of sepsis, which can progress to multiorgan failure [2]. Given the complex and interconnected nature of these conditions, early diagnosis and prompt intervention are critical to preventing further deterioration and improving patient outcomes. Fortunately, AI has made significant advancements in addressing these challenges, offering innovative tools for early diagnosis and management to improve patient outcomes.

### Sepsis diagnostics and drug treatment

Sepsis is defined as a life-threatening condition due to a dysregulated immune response to infection [31] and is a major complication within burn patients, posing itself as the leading cause of mortality following burn injury [32]. Symptomatically, the patients show signs of mental decline, systemic infection, irregular temperature, and critical illness that leads to septic shock and eventually to major organ dysfunction and failure [31]. Despite advances in burn care, the early identification and treatment of sepsis remain a difficult challenge for clinicians.

In 2020, Tran *et al*. developed an ML platform designed to automatically produce ML models that could predict burn sepsis. They compared this approach, entitled Machine Intelligence Learning Optimizer (MILO), against their previously developed “traditional” ML-based programs and standard statistical methods [33]. MILO generated a K-nearest neighbor (KNN) model, which, through five clinical markers, was able to achieve a 90% accuracy in sepsis identification, with a receiver operating characteristic area under the curve (ROC-AUC) of 0.96, in a sample of 211 adult burn patients. This proof-of-concept tool requires further validation across multicenter studies to affirm its efficacy; however, this suggests promise in AI-enhanced tools for diagnosing burn-induced sepsis and should encourage a wider research interest. Besides burn-related sepsis, there are multiple studies that have explored the use of AI in optimizing fluid management for sepsis patients. For example, Zhang *et al*. in 2024 employed ML algorithms integrated with multi-omics data to stratify septic shock patients into distinct subgroups. This classification aimed to tailor fluid resuscitation strategies, thereby potentially enhancing treatment outcomes [34]. In another study, a reinforcement learning algorithm coupled with a neural network was utilized to evaluate a dynamic treatment regimen for the fluid resuscitation in sepsis patients, enabling the development of patient-specific fluid management strategies [35].

Antibiotic administration is a key aspect of sepsis treatment. Indeed, aminoglycoside antibiotic treatments are commonly provided to burn patients with sepsis to manage Gram-negative infections [36]. Unfortunately, aminoglycosides have a narrow therapeutic range, with one study finding that the serum concentration of aminoglycosides in burn patients could not reach a concentration sufficient to induce a therapeutic effect [37]. Identifying the optimal dose within patients is a difficult task for clinicians and requires further investigations into its pharmacokinetic properties. Yamamura *et al*. addressed this issue by developing an artificial neural network (ANN) model that aimed to predict arbekacin plasma concentration via burn severity and identify patients who will experience subtherapeutic concentrations based on various physiological parameters [38]. After goodness of fit evaluation by the square root of the sum of squares and Akaike’s information criteria, the weight between neurons was optimized by the conjugate gradient descent method during training of the model. The authors concluded that, when compared to predictions made by a linear model, the ANN demonstrated greater performance in predicting arbekacin plasma concentration based on dose, body mass index, parenteral fluid, creatinine concentration, and burn area (excluding area of skin graft). Moreover, the results indicate not only that arbekacin’s pharmacokinetic properties are influenced by physiological measures altered postburn, but that AI models can be utilized to improve personalized dosing and overall efficacy of aminoglycoside antibiotics in burn patients. The same group also presented the use of another ANN model to predict the response of arbekacin against methicillin-resistant *Staphylococcus aureus* infection in burn patients [39]. Once again, the ANN displayed a superior performance in predicting the arbekacin response based on the area of the burn after skin grafting, when compared to a logistic regression (LR) model. While this model will require further testing within larger sample sizes before it reaches a performance level suitable for clinical application, there is potential that this AI model could help inform clinicians on whether patients are more likely to respond positively or poorly to antibiotic treatment. Although the aforementioned studies did not directly examine sepsis in burn patients, they addressed infections in this population, which are likely to progress into sepsis. Treatment with aminoglycosides will help prevent the development of sepsis following burn-related infection. Unfortunately, few studies have explored the application of AI in burn-associated sepsis; however, this scarcity should not discourage further investigation, as existing evidence highlights the promising potential of AI to enhance sepsis diagnosis and treatment.

### Inhalation and kidney injuries

There is a high incidence of inhalation injury (II) in patients with severe burn injury, a troubling statistic for clinicians given the complications and detrimental outcomes associated with II. In some cases, it is the leading cause of death in burn patients [40]. Early diagnosis of II in burn patients is crucial for the timely implementation of a treatment strategy to mitigate the risk of death. Clinicians use multiple signs and symptoms to diagnose II upon admission, such as facial burns, soot in the upper respiratory tract, singed nasal hair, a smoky smell, respiratory distress, and a bronchoscopy examination [41]. In 2023, an AI tool was used to assess the severity and presence of II by utilizing a large retrospective dataset of 341 burn patients with suspected II based on bronchoscopy diagnosis data. In this study, the authors developed two gradient boosting (GBM) models that utilized various physiological parameters as input features. Model 1 was designed to predict whether the patients had mild or severe inhalation injuries, whereas model 2 was used to predict the absence or presence of II [42]. Model 1 demonstrated “excellent” discrimination with an AUC of 0.883, identifying subjective inhalation reminiscence, soot in the upper airway, burn injury occurring in an enclosed space, elevated carboxyhemoglobin (COHB) level, and singed nasal vibrissae as the top five distinguishing risk factors between mild and severe II. Model 1 also determined that the incidence of pneumonia and mortality was significantly higher in patients with severe II. In contrast, model 2 showed “acceptable” discrimination based on an AUC of 0.862, identifying singed nasal vibrissae, soot in the upper airway, burn depth, systolic blood pressure at triage, and elevated COHB level as the top five distinguishing risk factors for the presence or absence of II. Similar to model 1, the second model found that the incidence of pneumonia, mortality, and duration of hospitalization was significantly higher in patients with II. Not only was this the first study to employ ML algorithms for the prediction of II, its severity, and associated outcomes, but the findings also demonstrate promise in predictive performance of ML models for these tasks and could greatly assist clinicians when bronchoscopies are not available immediately upon admission. However, before clinical integration of an AI tool for improved II diagnosis and assessment can be considered, further studies with larger sample sizes are needed to validate these findings and provide a stronger rationale for implementation in routine practice.

In addition to II, acute kidney injury (AKI) is another common complication seen in patients with severe burn injury, affecting up to 58% of this population [43, 44]. AKI is most common during the first week postburn as it develops in response to inadequate resuscitation [44, 45]. Unfortunately, early diagnosis remains an issue as creatinine, a readily used diagnostic marker for AKI, has limitations owing to its slow half-life [46] and high biological variability [47]. Urine output (UOP) is commonly paired with creatinine to aid in AKI diagnosis; however, it too has limitations, as in critically ill patients, who experience decreased glomerular filtration rate and may still present with unchanged UOP [48]. Moreover, an investigation led by Tran *et al.* sought to determine the predictive ability of ML in diagnosing burn-induced AKI through both traditional (creatinine and UOP) and novel markers of AKI, such as neutrophil gelatinase-associated lipocalin (NGAL) and N-terminal B-type natriuretic peptide (NT-proBNP) [33]. NGAL, UOP, creatinine, and NT-proBNP were measured within 24 h postburn from 50 adult burn patients and used to train ML models based on the KNN algorithm. The results found that models using UOP, creatinine, and NT-proBNP achieved up to 90% accuracy in predicting AKI, whereas models using only NT-proBNP and creatinine demonstrated an 85%–90% accuracy. According to the Kidney Disease: Improving Global Outcomes (KDIGO) criteria—which incorporate plasma creatinine and UOP measurements—it took an average of 42.7 h after admission to establish an AKI diagnosis in these patients. In contrast, the aforementioned ML models took an average of only 18.8 h to identify AKI. Another key finding from this study was the near 100% accuracy in predicting AKI when using NGAL as the sole input measure within the ML algorithm. This corroborates previous findings, which have identified statistically significant increases in NGAL at 4 h postburn to be predictive of AKI [49, 50]. Not only do these results highlight the predictive potential of NGAL, but they suggest that, even in its absence, ML models can enhance the prediction and diagnosis of burn-induced AKI using traditional clinically available markers. Indeed, a year later, the same group built on these findings by applying this ML approach, as well as others, including LR, support vector machine (SVM), random forest (RF), and deep neural network (DNN), to prospectively identify AKI in 51 adult patients with severe burns and other non-burn traumas. As per their previous work, measurements of included biomarkers were done within 24 h of the injury. The DNN and LR models, using NGAL and NT-proBNP, demonstrated the strongest results achieving a 92% generalization accuracy, 91% sensitivity, 93% specificity, and an AUC of 92%, a performance nearly emulated by the LR model which used creatinine rather than NT-proBNP and had a 90% accuracy, 91% sensitivity, 90% specificity, and an AUC of 91%. When compared with the best-performing model that excluded NGAL—an RF model using creatinine and UOP, which achieved 71% accuracy, 82% sensitivity, 68% specificity, and an AUC of 0.75—it becomes evident that NGAL holds significant value as a predictive biomarker for AKI. The ML models once again demonstrated timely predictions, identifying AKI an average of 32.5 h (2.5 days) before patients met KDIGO criteria [51]. These studies provide strong support for the incorporation of NGAL as a diagnostic measure for AKI as well as demonstrate the potential for AI to greatly enhance the speed and accuracy of AKI diagnosis following burn injury.

### Postburn outcomes

After exploring AI’s role in assessing wound closure and diagnosing burn-related complications, it is essential to consider the broader impact of burn injuries on both patients and the healthcare system. Mortality and length of hospital stay are two of the most significant outcomes, influencing not only survival but also long-term recovery, resource allocation, and healthcare costs. Predicting these outcomes with greater accuracy can help guide clinical decisions, optimize patient care, and improve hospital stays. AI-driven models have shown promise in leveraging vast datasets to identify key predictors of mortality and hospitalization duration, offering valuable insights for early intervention and personalized treatment strategies.

### Mortality prediction

Severe burn injuries significantly increase one’s risk of mortality due to numerous factors, such as sepsis, septic shock, organ failure, significant protein and lipid catabolism, and profound inflammatory activation [52, 53]. Given the complex and multifactorial nature of burn injuries, along with the numerous factors contributing to adverse outcomes, clinicians often face challenges in developing an effective treatment plan that can reduce the risk of burn-related mortality. Moreover, despite advances in statistical techniques, like the use of burn size to predict mortality, there remains a need to improve early risk identification in burn patients to help improve the intervention strategy and reduce the number of deaths in burn clinics.

Evidently, an AI model that rapidly analyzes multiple clinical measures and accurately predicts one’s risk of mortality within the acute stage following burn injury could prove instrumental in reshaping patient outcomes. Patil *et al*. used multiple data mining algorithms to predict mortality of burn patients through age, sex, and %TBSA burned in eight different parts of the body as features from 180 burn patients [54]. Data mining is an innovative technique, harnessing ML models to uncover key patterns and associations within large datasets. Out of the several models tested, including Naive Bayes (NB), decision tree (DT), SVM, and back propagation, NB proved to be the best predictor of mortality with 97.78% accuracy. Another investigation attempted to predict mortality within a large burn patient population using ML methods such as an ANN, SVM, RF, and NB models. The performance of these algorithms was then compared to an established logistic mortality model, with all methods incorporating features such as age, %TBSA burned, presence of II, three or more existing disorders, and type of burn [55]. The authors found no clinically relevant difference in performance between the established logistic mortality model and ML methods, as the AI models had comparable discriminatory abilities, sensitivities, specificities, and PPVs. Another study conducted by Fransén *et al*. in 2018 assessed the ability of ML algorithms to predict mortality using intensive care data from burn patients. The authors compared the performance of multiple ML models [DT, extreme boosting, RF, SVM, and a generalized linear regression (LR) model] to the Baux score and revised Baux score, common mortality prediction tools for burn injury. Using 17 prognostic variables selected from 92 patients, it was found that the ML algorithms performed comparably in predicting mortality, as there were no statistically significant differences between them and the Baux score and revised Baux score [56].

An additional investigation used only six clinical measures (TBSA, II, full-thickness burn, age, gender, and burn types) as input features for six ML models (KNN, DT, RF, SVM, Multi-Layer Perceptron, and AdaBoost) and assessed their ability to accurately predict mortality out of 363 burn patients. From these algorithms, AdaBoost displayed the strongest performance in predicting mortality, achieving an accuracy of 90% with an AUC of 92% [57]. While the study did not compare against presently used prediction tools, as done in the latter study, the findings demonstrate that ML models equipped with the right input features can have positive benefits within clinical practice for determining risk of mortality. Finally, a robust single-center study aimed to compare the predictive performance of their proposed ANN model against 32 ML models when considering 40 patient parameters (demographic and biochemical data) collected on the first day postburn. The authors found that the ANN model outperformed all ML models, demonstrating an accuracy of 95.92%, an impressive feat given the volume of parameters included [58]. Overall, there appears to be promise in AI-based tools for mortality prediction in burn patients; however, it is clear that larger studies and further refinement of these algorithms must be done before this technology can be incorporated into a clinical setting.

### Length of hospital stay prediction

Patients with severe burns experience extended hospitalization times as treatment and wound care are often extensive and time-consuming. Hospitalization times serve not only as an indicator of injury-related morbidity and the incidence of clinical complications [59, 60], but also directly influence the management and allocation of resources, as well as treatment costs [61, 62]. In 1986, it was published that %TBSA burned can be used as a rough estimate to predict LOS, with 1% TBSA amounting to 1 day of stay [63]. Despite validation [64] and general acceptance of this rule, concerns have arisen regarding its oversimplification and the need for techniques that provide greater precision in predicting LOS [63, 65, 66]. In 2021, Elrod *et al.* sought to address this gap by comparing the aforementioned rule of thumb against two AI models designed to predict LOS following burn injury using parameters such as TBSA itemized by degree of burn, age, gender, II, and cause of injury [59]. Linear regression (LR) and RF algorithms were used and tested on 8542 datasets of pediatric burn patients provided by the German Burn Registry, and all three models were validated by the k-fold cross-validation method to estimate the generalization error in future or real-world use. Through their analysis, which depicted the expected difference between each predictive model if used on new real-world data, the authors concluded that both AI models significantly outperformed the rule of thumb. Additionally, prediction error increased across all three methods with increasing TBSA and the presence of full-thickness burns. This is most likely due to the complexity of wound treatment required, as well as the heightened risk of MOF and sepsis. In another investigation, four ML models, namely LR, RF, LightGBM, and XBGBoost were tested on their ability to predict prolonged hospital stay (hospitalization >14 days) using 12 clinical parameters comprising demographic (age, gender, etc.), burn (TBSA and burn site), and laboratory data (white blood cells (WBC), hemoglobin, creatinine, and glutamate pyruvate transaminase) [67]. Despite these results being limited by an absence of comparison to the rule of thumb, the RF algorithm demonstrated strong performance with an accuracy of 79%, sensitivity of 80%, specificity of 79%, and an AUC of 80.1%, as did the XBGBoost (78% accuracy, 80% sensitivity, 76% specificity, and an AUC of 81.5%). Overall, these findings highlight the benefit of AI-based models for LOS prediction, which can incorporate multiple measures beyond %TBSA burned to enhance precision and accuracy. Further testing of such algorithms is required before clinical implementation; however, if an LOS predictive tool was available for clinicians, it could provide immediate help in counseling patient families with expectations of care as well as improve management of hospital resources.

### The use of clinical data and ethical considerations in artificial intelligence applications

While AI is emerging as a promising tool in the field of burn care, offering innovative approaches for diagnosis, treatment, and outcome prediction, it is crucial to address the broader implications of using clinical data within AI platforms. The integration of sensitive patient information raises significant ethical concerns, particularly those related to data privacy, consent, and the potential for bias. It is essential to establish clear guidelines for how clinical data will be shared, accessed, and utilized in AI systems to ensure that patient rights are respected and protected and that these technologies are used responsibly and transparently. It is recommended that AI development teams review and ensure compliance with privacy legislation such as the General Data Protection Regulation, the Personal Information Protection and Electronic Documents Act, and the Health Insurance Portability and Accountability Act, as well as consult with data protection specialists [4]. As AI continues to evolve in the medical field, thoughtful consideration of these ethical issues will be key to maintaining trust and safeguarding patient well-being.

**Figure 3:**
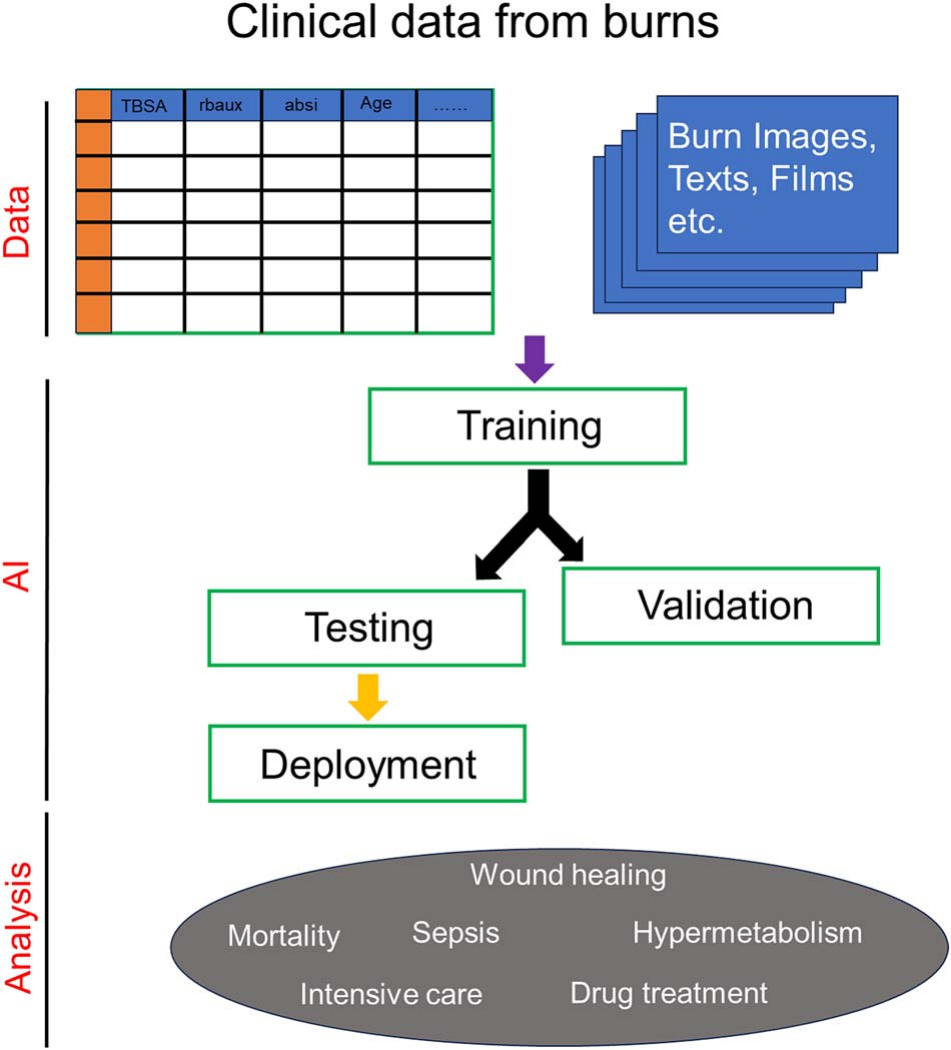
Clinical data and workflow of AI; the data from the burn patient can be structured with numerical values for machine learning and unstructured data like images, texts, films, etc., for deep learning; these data are cleaned and trained using specific algorithms; then, validation and testing processes are performed; at the final stage, the AI tool is deployed for clinical use

Here, we briefly outline the workflow of how clinical data is used in AI applications. Initially, a digital health record system in a hospital keeps all the clinical data of patients. These datasets are freely available to researchers upon agreement for the study through an archiving platform [68]. In addition, approval of the use of data for the specific study is taken from the institution where it is carried out, and written consent is obtained from each individual patient. All the participating patients are deidentified to protect privacy before the start of the study. A typical clinical dataset of burns comprises medical history, laboratory tests, vitals, wound images, TBSA, Baux score, Abbreviated Burn Severity Index (ABSI) score, charts, notes and reports, II, comorbidities, demographics, images, texts, films, etc. All these data are selected as variables and listed in a spreadsheet as a structured matrix for the analysis. Each variable can be either a numerical value or a categorical value and is used for the predictive analysis. Often, the clinical numerical data are used for the ML algorithms, whereas burn images, texts, films, etc., are used for the DL algorithms (Figure 3).

### Future outlook: integrating high-throughput multi-omics approaches with artificial intelligence

**Figure 4:**
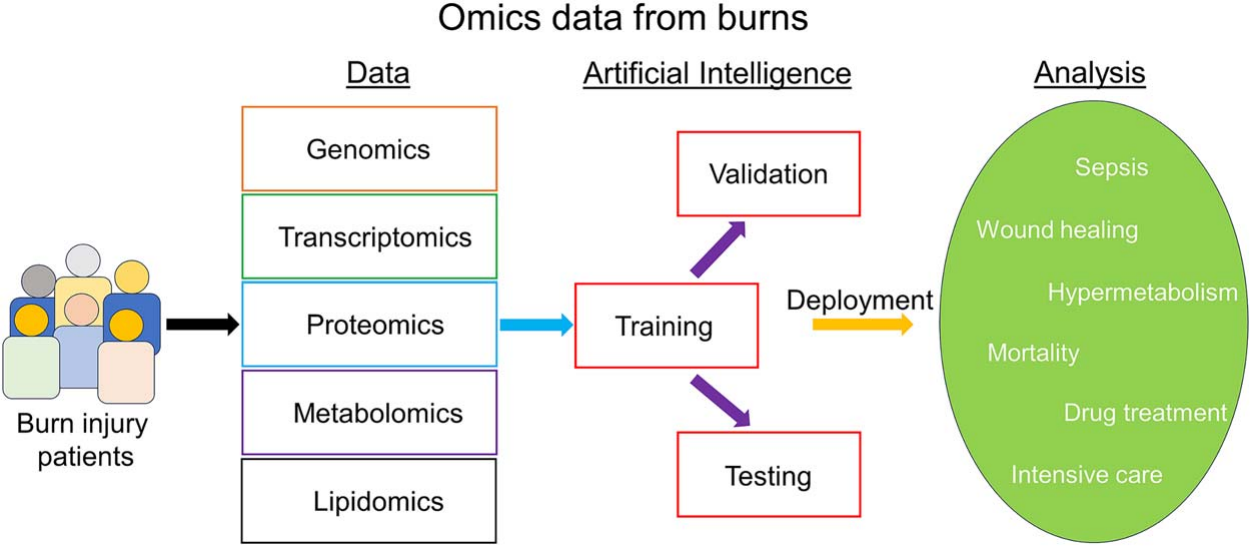
Omics data and AI; the data generated from genomics, transcriptomics, proteomics, metabolomics, lipidomics, etc., using burn patient samples, are taken as input for the machine learning or deep learning (DL); these omics data are trained by specific algorithms and validated; testing is done, and new data are used for the predictive or classification analysis and discovery

Now, we turn our attention to the “bench to bedside” framework, which serves as the foundation of much scientific research and clinical application. This review has thus far focused on the bedside aspect, exploring how AI can be leveraged to directly benefit burn patients. However, a growing body of research is taking place behind the scenes, where efforts are directed at uncovering the mechanisms driving disease progression and identifying targeted therapeutics that can improve patient outcomes. In this context, high-throughput technologies such as omics are playing an increasingly important role. By generating vast amounts of data on genomics, proteomics, and metabolomics, these approaches, when integrated with AI, can significantly enhance our understanding of disease at a molecular level and thereby improve clinical practice (Figure 4). Moreover, studies are now showing the efficacy of combined ML and omics approaches for the discovery of novel biomarkers of disease [69–72], as well as in the improvement of diagnostic accuracy [73, 74]. Omics datasets are inexplicably large, and while non-AI analytical approaches have yielded positive results, the power of ML models is seemingly complementary to handle the complex nature of omics data, further uncover patterns and trends otherwise difficult to identify by conventional means. Unfortunately, there is limited evidence on the combined AI and omics approach within burn research. Nonetheless, we believe this synergy of cutting-edge research and AI offers immense potential to revolutionize both basic science and clinical burn care.

## Conclusions

Burn injuries pose a unique challenge due to the difficulty in accurately assessing burn severity and wound healing, as well as limitations in early prediction of burn-related complications like sepsis, AKI, and II. While burn care has improved greatly over the last decades, as evidenced by a declining mortality rate, our efforts to enhance and transform this aspect of burn care must improve if we are to sustain this trend and reduce the negative impact of burn injury. To address these limitations, the current needs of burn care necessitate the introduction of emerging technologies such as AI. Herein, we have highlighted some of the most effective models evidenced in the literature for each AI application in burns care. Moreover, the most frequently tested AI application in burn research was mortality prediction. As the development of these innovative techniques persists, and subsequent evidence continues to amount regarding its efficacy from randomized clinical trials for generalizable to clinical practice, we may begin to see the incorporation of these tools within burn clinics globally.

## References

[1] Yakupu A, Zhang J, Dong W, Song F, Dong J, Lu S. The epidemiological characteristic and trends of burns globally. *BMC Public Health* 2022;22:1596. 10.1186/s12889-022-13887-2. 35996116 PMC9396832

[2] Jeschke MG, van Baar ME, Choudhry MA, Chung KK, Gibran NS, Logsetty S. Burn injury. *Nat Rev Dis Primers* 2020;6:11. 10.1038/s41572-020-0145-5. 32054846 PMC7224101

[3] Greener JG, Kandathil SM, Moffat L, Jones DT. A guide to machine learning for biologists. *Nat Rev Mol Cell Biol* 2022;23:40–55. 10.1038/s41580-021-00407-0. 34518686

[4] de Hond AAH, Leeuwenberg AM, Hooft L, Kant IMJ, Nijman SWJ, van Os HJA. et al. Guidelines and quality criteria for artificial intelligence-based prediction models in healthcare: a scoping review. *NPJ Digit Med* 2022;5:2. 10.1038/s41746-021-00549-7. 35013569 PMC8748878

[5] E Moura FS, Amin K, Ekwobi C. Artificial intelligence in the management and treatment of burns: a systematic review. Burns Trauma 2021;9:tkab022. 10.1093/burnst/tkab022. 34423054 PMC8375569

[6] Liu Y, Chen P-HC, Krause J, Peng L. How to read articles that use machine learning: users’ guides to the medical literature. *JAMA* 2019;322:1806–16. 10.1001/jama.2019.16489. 31714992

[7] AbdelWahab ME, Sadaka MS, Elbana EA, Hendy AA. Evaluation of prognostic factors affecting length of stay in hospital and mortality rates in acute burn patients. *Ann Burns Fire Disasters* 2018;31:83–8. 30374257 PMC6199018

[8] Kraft R, Herndon DN, Al-Mousawi AM, Williams FN, Finnerty CC, Jeschke MG. Burn size and survival probability in paediatric patients in modern burn care: a prospective observational cohort study. *Lancet* 2012;379:1013–21. 10.1016/S0140-6736(11)61345-7. 22296810 PMC3319312

[9] Jeschke MG, Pinto R, Kraft R, Nathens AB, Finnerty CC, Gamelli RL. et al. Morbidity and survival probability in burn patients in modern burn care. *Crit Care Med* 2015;43:808–15. 10.1097/CCM.0000000000000790. 25559438 PMC4359665

[10] Wallace AB . The exposure treatment of burns. *Lancet* 1951;1:501–4. 10.1016/s0140-6736(51)91975-7. 14805109

[11] García-Ballesteros DI, Rivera-Martínez DDC, García-Pérez MM, Valdés-Flores E, Castro-Govea Y, Chacón-Moreno HJ. Evaluation and optimization of the Wallace rule of nines for the estimation of total body surface area in obese and nonobese populations. *J Emerg Med* 2023;65:e320–7. 10.1016/j.jemermed.2023.05.017. 37709577

[12] Chang CW, Lai F, Christian M, Chen YC, Hsu C, Chen YS. et al. Deep learning–assisted burn wound diagnosis: diagnostic model development study. JMIR Med Inform 2021;9:e22798. 10.2196/22798. 34860674 PMC8686480

[13] Harish V, Raymond AP, Issler AC, Lajevardi SS, Chang L-Y, Maitz PKM. et al. Accuracy of burn size estimation in patients transferred to adult burn units in Sydney, Australia: an audit of 698 patients. *Burns* 2015;41:91–9. 10.1016/j.burns.2014.05.005. 24972983

[14] Chang CW, Ho CY, Lai F, Christian M, Huang SC, Chang DH. et al. Application of multiple deep learning models for automatic burn wound assessment. *Burns* 2023;49:1039–51. 10.1016/j.burns.2022.07.006. 35945064

[15] Xu X, Bu Q, Xie J, Li H, Xu F, Li J. On-site burn severity assessment using smartphone-captured color burn wound images. *Comput Biol Med* 2024;182:109171. 10.1016/j.compbiomed.2024.109171. 39362001

[16] Evers LH, Bhavsar D, Mailänder P. The biology of burn injury. *Exp Dermatol* 2010;19:777–83. 10.1111/j.1600-0625.2010.01105.x. 20629737

[17] Jaskille AD, Shupp JW, Jordan MH, Jeng JC. Critical review of burn depth assessment techniques: part I. Historical review. *J Burn Care Res* 2009;30:937–47. 10.1097/BCR.0b013e3181c07f21. 19898102

[18] Monstrey S, Hoeksema H, Verbelen J, Pirayesh A, Blondeel P. Assessment of burn depth and burn wound healing potential. *Burns* 2008;34:761–9. 10.1016/j.burns.2008.01.009. 18511202

[19] Jaspers MEH, van Haasterecht L, van Zuijlen PPM, Mokkink LB. A systematic review on the quality of measurement techniques for the assessment of burn wound depth or healing potential. *Burns* 2019;45:261–81. 10.1016/j.burns.2018.05.015. 29941159

[20] Thatcher JE, Squiers JJ, Kanick SC, King DR, Lu Y, Wang Y. et al. Imaging techniques for clinical burn assessment with a focus on multispectral imaging. *Adv Wound Care (New Rochelle)* 2016;5:360–78. 10.1089/wound.2015.0684. 27602255 PMC4991589

[21] Resch TR, Drake RM, Helmer SD, Jost GD, Osland JS. Estimation of burn depth at burn centers in the United States: a survey. *J Burn Care Res* 2014;35:491–7. 10.1097/BCR.0000000000000031. 25144808

[22] Thatcher JE, Yi F, Nussbaum AE, DiMaio JM, Dwight J, Plant K. et al. Clinical investigation of a rapid non-invasive multispectral imaging device utilizing an artificial intelligence algorithm for improved burn assessment. *J Burn Care Res* 2023;44:969–81. 10.1093/jbcr/irad051. 37082889 PMC10321393

[23] Lee JJ, Abdolahnejad M, Morzycki A, Freeman T, Chan H, Hong C. et al. Comparing artificial intelligence guided image assessment to current methods of burn assessment. *J Burn Care Res* 2024;**46**:6–13. 10.1093/jbcr/irae121. PMC11761738 38918900

[24] Khalaf F, Barayan D, Saldanha S, Jeschke, MG. Metabolaging: a new geroscience perspective linking aging pathologies and metabolic dysfunction. Metab Clin Exp 2025;166:156158. 10.1016/j.metabol.2025.156158. 39947519

[25] Singer AJ, Boyce ST. Burn wound healing and tissue engineering. *J Burn Care Res* 2017;38:e605–13. 10.1097/BCR.0000000000000538. 28328668 PMC5461657

[26] Xiao-Wu W, Herndon DN, Spies M, Sanford AP, Wolf SE. Effects of delayed wound excision and grafting in severely burned children. *Arch Surg* 2002;137:1049–54. 10.1001/archsurg.137.9.1049. 12215159

[27] Kirn DS, Luce EA. Early excision and grafting versus conservative management of burns in the elderly. *Plast Reconstr Surg* 1998;102:1013–7. 10.1097/00006534-199809040-00013. 9734417

[28] Saaiq M, Zaib S, Ahmad S. Early excision and grafting versus delayed excision and grafting of deep thermal burns up to 40% total body surface area: a comparison of outcome. *Ann Burns Fire Disasters* 2012;25:143–7. 23467391 PMC3575152

[29] Ong YS, Samuel M, Song C. Meta-analysis of early excision of burns. *Burns.* 2006;32:145–50. 10.1016/j.burns.2005.09.005. 16414197

[30] Ethier O, Chan HO, Abdolahnejad M, Morzycki A, Fansi Tchango A, Joshi R. et al. Using computer vision and artificial intelligence to track the healing of severe burns. *J Burn Care Res* 2024;45:700–8. 10.1093/jbcr/irad197. 38126807

[31] Singer M, Deutschman CS, Seymour CW, Shankar-Hari M, Annane D, Bauer M. et al. The third international consensus definitions for sepsis and septic shock (Sepsis-3). *JAMA* 2016;315:801. 10.1001/jama.2016.0287. 26903338 PMC4968574

[32] Greenhalgh DG, Saffle JR, Holmes JH, Gamelli RL, Palmieri TL, Horton JW. et al. American burn association consensus conference to define sepsis and infection in burns. *J Burn Care Res* 2007;28:776–90. 10.1097/BCR.0b013e3181599bc9. 17925660

[33] Tran NK, Albahra S, Pham TN, Holmes JH, Greenhalgh D, Palmieri TL. et al. Novel application of an automated-machine learning development tool for predicting burn sepsis: proof of concept. *Sci Rep* 2020;10:12354. 10.1038/s41598-020-69433-w. 32704168 PMC7378181

[34] Zhang Z, Chen L, Sun B, Ruan Z, Pan P, Zhang W. et al. Identifying septic shock subgroups to tailor fluid strategies through multi-omics integration. *Nat Commun* 2024;15:9028. 10.1038/s41467-024-53239-9. 39424794 PMC11489719

[35] Liang W, Jia J. Reinforcement learning using neural networks in estimating an optimal dynamic treatment regime in patients with sepsis. *Comput Methods Prog Biomed* 2025;266:108754. 10.1016/j.cmpb.2025.108754. 40222267

[36] Nunez Lopez O, Cambiaso-Daniel J, Branski LK, Norbury WB, Herndon DN. Predicting and managing sepsis in burn patients: current perspectives. *Ther Clin Risk Manag* 2017;13:1107–17. 10.2147/TCRM.S119938. 28894374 PMC5584891

[37] Polk RE, Mayhall CG, Smith J, Hall G, Kline BJ, Swensson E. et al. Gentamicin and tobramycin penetration into burn eschar. Pharmacokinetics and microbiological effects. *Arch Surg* 1983;118:295–302. 10.1001/archsurg.1983.01390030027005. 6824430

[38] Yamamura S, Kawada K, Takehira R, Nishizawa K, Katayama S, Hirano M. et al. Artificial neural network modeling to predict the plasma concentration of aminoglycosides in burn patients. *Biomed Pharmacother* 2004;58:239–44. 10.1016/j.biopha.2003.12.012. 15183849

[39] Yamamura S, Kawada K, Takehira R, Nishizawa K, Katayama S, Hirano M. et al. Prediction of aminoglycoside response against methicillin-resistant *Staphylococcus aureus* infection in burn patients by artificial neural network modeling. *Biomed Pharmacother* 2008;62:53–8. 10.1016/j.biopha.2007.11.004. 18083323

[40] Shirani KZ, Pruitt BA, Mason AD. The influence of inhalation injury and pneumonia on burn mortality. *Ann Surg* 1987;205:82–7. 10.1097/00000658-198701000-00015. 3800465 PMC1492872

[41] Deutsch CJ, Tan A, Smailes S, Dziewulski P. The diagnosis and management of inhalation injury: an evidence based approach. *Burns.* 2018;44:1040–51. 10.1016/j.burns.2017.11.013. 29398078

[42] Yang S-Y, Huang C-J, Yen C-I, Kao Y-C, Hsiao Y-C, Yang J-Y. et al. Machine learning approach for predicting inhalation injury in patients with burns. *Burns.* 2023;49:1592–601. 10.1016/j.burns.2023.03.011. 37055284 PMC10032063

[43] Chen B, Zhao J, Zhang Z, Li G, Jiang H, Huang Y. et al. Clinical characteristics and risk factors for severe burns complicated by early acute kidney injury. *Burns* 2020;46:1100–6. 10.1016/j.burns.2019.11.018. 31839503

[44] Clark AT, Li X, Kulangara R, Adams-Huet B, Huen SC, Madni TD. et al. Acute kidney injury after burn: a cohort study from the parkland burn intensive care unit. *J Burn Care Res* 2019;40:72–8. 10.1093/jbcr/iry046. 30189043 PMC6300394

[45] Palmieri T, Lavrentieva A, Greenhalgh DG. Acute kidney injury in critically ill burn patients. Risk factors, progression and impact on mortality. *Burns* 2010;36:205–11. 10.1016/j.burns.2009.08.012. 19836141

[46] Watkins TR . The comprehensive community mental health center as a field placement for graduate social work students. *Community Ment Health J* 1975;11:27–32. 10.1007/BF01420461. 1132219

[47] Reinhard M, Erlandsen EJ, Randers E. Biological variation of cystatin C and creatinine. *Scand J Clin Lab Invest* 2009;69:831–6. 10.3109/00365510903307947. 19929276

[48] Legrand M, Payen D. Understanding urine output in critically ill patients. *Ann Intensive Care* 2011;1:13. 10.1186/2110-5820-1-13. 21906341 PMC3224471

[49] Howell E, Sen S, Palmieri T, Godwin Z, Bockhold J, Greenhalgh D. et al. Point-of-care B-type natriuretic peptide and neutrophil gelatinase-associated lipocalin measurements for acute resuscitation: a pilot study. *J Burn Care Res* 2015;36:e26–33. 10.1097/BCR.0000000000000098. 25188271

[50] Sen S, Godwin ZR, Palmieri T, Greenhalgh D, Steele AN, Tran NK. Whole blood neutrophil gelatinase-associated lipocalin predicts acute kidney injury in burn patients. *J Surg Res* 2015;196:382–7. 10.1016/j.jss.2015.03.033. 25890435 PMC4447708

[51] Rashidi HH, Sen S, Palmieri TL, Blackmon T, Wajda J, Tran NK. Early recognition of burn- and trauma-related acute kidney injury: a pilot comparison of machine learning techniques. *Sci Rep* 2020;10:205. 10.1038/s41598-019-57083-6. 31937795 PMC6959341

[52] Bloemsma GC, Dokter J, Boxma H, Oen IMMH. Mortality and causes of death in a burn centre. *Burns* 2008;34:1103–7. 10.1016/j.burns.2008.02.010. 18538932

[53] Hasan JA, Eriby QH, Hammoodi SA-K. Causes of mortality in burns, prospective study. International journal of surgery. *Science* 2019;3:261–4. 10.33545/surgery.2019.v3.i3e.178.

[54] Patil BM, Joshi RC, Toshniwal D, Biradar S. A new approach: role of data Mining in Prediction of survival of burn patients. *J Med Syst* 2011;35:1531–42. 10.1007/s10916-010-9430-2. 20703764

[55] Stylianou N, Akbarov A, Kontopantelis E, Buchan I, Dunn KW. Mortality risk prediction in burn injury: comparison of logistic regression with machine learning approaches. *Burns* 2015;41:925–34. 10.1016/j.burns.2015.03.016. 25931158

[56] Fransén J, Lundin J, Fredén F, Huss F. A proof-of-concept study on mortality prediction with machine learning algorithms using burn intensive care data. *Scars Burn Heal* 2022;8. 10.1177/20595131211066585. PMC8859689 35198237

[57] Yazıcı H . The effect of well-known burn-related features on machine learning algorithms in burn patients’ mortality prediction. Turkish journal of trauma and emergency. *Surgery* 2023;**29**:1130–7. 10.14744/tjtes.2023.79968. PMC10644077 37791433

[58] Çinar MA, Ölmez E, Erkiliç A, Bayramlar K, Er O. Machine learning models for early prediction of mortality risk in patients with burns: a single center experience. *J Plast Reconstr Aesthet Surg* 2024;89:14–20. 10.1016/j.bjps.2023.11.048. 38118361

[59] Elrod J, Mohr C, Wolff R, Boettcher M, Reinshagen K, Bartels P. et al. Using artificial intelligence to obtain more evidence? Prediction of length of hospitalization in Pediatric burn patients. *Front Pediatr* 2020;8:613736. 10.3389/fped.2020.613736. 33537267 PMC7849450

[60] Hussain A, Dunn KW. Predicting length of stay in thermal burns: a systematic review of prognostic factors. *Burns* 2013;39:1331–40. 10.1016/j.burns.2013.04.026. 23768707

[61] Gravante G, Montone A, Esposito G. Length of hospitalization: an important parameter for burned patients. *J Burn Care Res* 2007;28:537–8. 10.1097/BCR.0b013e318053db44. 17438495

[62] Jiménez R, López L, Dominguez D, Fariñas H. Difference between observed and predicted length of stay as an indicator of inpatient care inefficiency. *Int J Qual Health Care* 1999;11:375–84. 10.1093/intqhc/11.5.375. 10561028

[63] Johnson LS, Shupp JW, Pavlovich AR, Pezzullo JC, Jeng JC, Jordan MH. Hospital length of stay—does 1% TBSA really equal 1 day? *J Burn Care Res* 2011;32:13–9. 10.1097/BCR.0b013e318204b3ab. 21131842

[64] Saffle JR, Davis B, Williams P. Recent outcomes in the treatment of burn injury in the United States: a report from the American burn association patient registry. *J Burn Care Rehabil* 1995;16:219–32. 10.1097/00004630-199505000-00002. 7673300

[65] Taylor SL, Sen S, Greenhalgh DG, Lawless M, Curri T, Palmieri TL. Not all patients meet the 1day per percent burn rule: a simple method for predicting hospital length of stay in patients with burn. *Burns* 2017;43:282–9. 10.1016/j.burns.2016.10.021. 28041754

[66] Tan T, Wong DSY. Is the target of 1 day of stay per 1% total body surface area burned achieved in chemical burns? *Ann Plast Surg* 2016;77:S39–42. 10.1097/SAP.0000000000000716. 26808735

[67] Yeh C-C, Lin Y-S, Chen C-C, Liu C-F. Implementing AI models for prognostic predictions in high-risk burn patients. *Diagnostics* 2023;13:2984. 10.3390/diagnostics13182984. 37761351 PMC10528558

[68] Johnson AEW, Bulgarelli L, Shen L, Gayles A, Shammout A, Horng S. et al. MIMIC-IV, a freely accessible electronic health record dataset. *Sci Data* 2023;10:1. 10.1038/s41597-022-01899-x. 36596836 PMC9810617

[69] She H, Du Y, Du Y, Tan L, Yang S, Luo X. et al. Metabolomics and machine learning approaches for diagnostic and prognostic biomarkers screening in sepsis. *BMC Anesthesiol* 2023;23:367. 10.1186/s12871-023-02317-4. 37946144 PMC10634148

[70] Fortino V, Wisgrill L, Werner P, Suomela S, Linder N, Jalonen E. et al. Machine-learning-driven biomarker discovery for the discrimination between allergic and irritant contact dermatitis. *Proc Natl Acad Sci USA* 2020;117:33474–85. 10.1073/pnas.2009192117. 33318199 PMC7776829

[71] Bifarin OO, Gaul DA, Sah S, Arnold RS, Ogan K, Master VA. et al. Machine learning-enabled renal cell carcinoma status prediction using multiplatform urine-based metabolomics. *J Proteome Res* 2021;20:3629–41. 10.1021/acs.jproteome.1c00213. 34161092 PMC9847475

[72] Shen X, Wang C, Liang N, Liu Z, Li X, Zhu Z-J. et al. Serum metabolomics identifies dysregulated pathways and potential metabolic biomarkers for hyperuricemia and gout. *Arthritis Rheumatol* 2021;73:1738–48. 10.1002/art.41733. 33760368

[73] Rong J, Sun G, Zhu J, Zhu Y, Chen Z. Combination of plasma-based lipidomics and machine learning provides a useful diagnostic tool for ovarian cancer. *J Pharm Biomed Anal* 2025;253:116559. 10.1016/j.jpba.2024.116559. 39514983

[74] Hsu N-W, Chou K-C, Wang Y-TT, Hung C-L, Kuo C-F, Tsai S-Y. Building a model for predicting metabolic syndrome using artificial intelligence based on an investigation of whole-genome sequencing. *J Transl Med* 2022;20:190. 10.1186/s12967-022-03379-7. 35484552 PMC9052619

